# The Role of Hippocampal NMDA Receptors in Long-Term Emotional Responses following Muscarinic Receptor Activation

**DOI:** 10.1371/journal.pone.0147293

**Published:** 2016-01-21

**Authors:** Alexandre A. Hoeller, Ana Paula R. Costa, Maíra A. Bicca, Filipe C. Matheus, Gilliard Lach, Francesca Spiga, Stafford L. Lightman, Roger Walz, Graham L. Collingridge, Zuner A. Bortolotto, Thereza C. M. de Lima

**Affiliations:** 1 Postgraduate Program in Medical Sciences, Center of Health Sciences, University Hospital, Federal University of Santa Catarina, Florianópolis, SC, 88040–970, Brazil; 2 Department of Pharmacology, Center of Biological Sciences, Federal University of Santa Catarina, Florianópolis, SC, 88040–970, Brazil; 3 Institute of Pharmacology, Innsbruck Medical University, Innsbruck, 6020, Austria; 4 Henry Wellcome Laboratories for Integrative Neuroscience and Endocrinology, School of Clinical Sciences, University of Bristol, Dorothy Hodgkin Building, Bristol, BS1 3NY, United Kingdom; 5 Department of Clinical Medicine, Center of Health Sciences, University Hospital, Federal University of Santa Catarina, Florianópolis, SC, 88040–970, Brazil; 6 Centre for Synaptic Plasticity, School of Physiology, Pharmacology and Neuroscience, University of Bristol, Dorothy Hodgkin Building, Bristol, BS1 3NY, United Kingdom; University of Modena and Reggio Emilia, ITALY

## Abstract

Extensive evidence indicates the influence of the cholinergic system on emotional processing. Previous findings provided new insights into the underlying mechanisms of long-term anxiety, showing that rats injected with a single systemic dose of pilocarpine—a muscarinic receptor (mAChR) agonist—displayed persistent anxiogenic-like responses when evaluated in different behavioral tests and time-points (24 h up to 3 months later). Herein, we investigated whether the pilocarpine-induced long-term anxiogenesis modulates the HPA axis function and the putative involvement of NMDA receptors (NMDARs) following mAChRs activation. Accordingly, adult male Wistar rats presented anxiogenic-like behavior in the elevated plus-maze (EPM) after 24 h or 1 month of pilocarpine injection (150 mg/kg, i.p.). In these animals, mAChR activation disrupted HPA axis function inducing a long-term increase of corticosterone release associated with a reduced expression of hippocampal GRs, as well as consistently decreased NMDAR subunits expression. Furthermore, in another group of rats injected with memantine–an NMDARs antagonist (4 mg/kg, i.p.)–prior to pilocarpine, we found inhibition of anxiogenic-like behaviors in the EPM but no further alterations in the pilocarpine-induced NMDARs downregulation. Our data provide evidence that behavioral anxiogenesis induced by mAChR activation effectively yields short- and long-term alterations in hippocampal NMDARs expression associated with impairment of hippocampal inhibitory regulation of HPA axis activity. This is a novel mechanism associated with anxiety-like responses in rats, which comprise a putative target to future translational studies.

## Introduction

Despite the close involvement of muscarinic ligands controlling cognitive and emotional processes [1–3], there are still critical questions regarding their precise physiological role, even taking into account their potential use in clinical trials [4]. We have previously shown that rats that do not convulse after a single injection of pilocarpine (75–350 mg/kg)–a non-selective muscarinic receptor (mAChR) agonist–presented anxiogenic-like behaviors when evaluated 24 h up to 3 months after the treatment, suggesting a new and useful experimental model for studying long-lasting anxiety [5–7]. Interestingly, acetylcholine modulates not only muscarinic but also glutamatergic postsynaptic synapses in several limbic areas [8, 9] resulting in both excitatory or inhibitory effects [10], besides the modulation of anxiety in rodents [11, 12] and humans [13, 14] as well as in the regulation of hormonal responses and stress-related behaviors [15, 16].

Acute stress may induce a transitory increase of plasmatic corticosterone (CORT), leading to an impaired inhibitory feedback of the hypothalamic-pituitary-adrenal (HPA) axis, which in turn induces augmented stress- and anxiogenic-like responses and also long-term modulation of the hippocampal glucocorticoid receptors (GRs) [17, 18]. The hippocampus is a major center for inhibition of the HPA axis and it has been shown to regulate the decrease of glucocorticoid release [19, 20] besides participating on the termination of stress responses [21, 22]. Cholinergic inputs from the basal forebrain can modulate hippocampal activity [23] which is significantly increased following stress [24] although the way in which cholinergic function converges to regulate hippocampal inputs in these responses is unclear.

There is growing evidence supporting the role of the glutamatergic system in the control of stress and affective disorders, mainly showing abnormal NMDAR transmission [25–30]. In rodents, acute stress largely increases extracellular levels of glutamate and aspartate in pivotal limbic brain areas involving affective-like behaviors [31] which are also attenuated when glucocorticoids are withdrawn after adrenalectomy [32]. Moreover, treatment with most of competitive and non-competitive NMDAR antagonists results in anxiolytic- and antidepressant-like effects when rats are evaluated in several behavioral tasks [33–35]. In this sense, there are no doubts regarding the involvement of NMDA signaling in controlling emotional states, although it remains unclear “if” or “how” this modulation may result from activation of other neurotransmitter systems, especially the muscarinic one.

Experimental studies investigating the involvement of mAChRs on modulation of behavioral anxiety in rodents have revealed that blockade of M1 mAChR subtypes produces anxiolytic-like effects [36, 37]. Furthermore, fear-related autonomic responses following the stimulation of amygdala nuclei are triggered by M1 subtypes [38]. Therefore, understanding the way in which NMDAR subunits are modulated after muscarinic activation in this situation may shed light on the mechanism of anxiogenesis, and ratify the muscarinic system as a potential target for treatment of emotional disorders.

## Material and Methods

### Animals

Adult male Wistar rats (2–3 months old, weighing 200–300 g) were housed in groups of four per cage and kept in a room with controlled temperature (22 ± 2°C) and a 12-h light/dark cycle (lights on at 07:00 a.m.) with free access to food and water, except during the experiments. Rats were allowed to adapt to the laboratory conditions for one week before the experiments. Behavioral experiments and hormonal dosages were carried out during the light phase of the cycle (between 13:00 and 18:00). All experiments were conducted in accordance with international standards of animal welfare recommended by the Brazilian Law (#11.794–10/08/2008) and Animals (Scientific Procedures) Act 1986, with experimental protocols approved by the Committee for Ethics in Animal Research of the Federal University of Santa Catarina (CEUA-UFSC #23080.025621/2009-03) and Home Office License 30/2512 (UK). The minimum number of animals and duration of observation required to obtain consistent data was used.

### Drugs and treatments

Pilocarpine hydrochloride (a non-selective muscarinic receptor agonist; Sigma-Aldrich Co., St. Louis, USA, 150mg/kg, i.p.) and memantine hydrochloride (an NMDA receptor antagonist; Ebix®, Merz Pharma GmbH & Co. KgaA, Frankfurt, Germany, 4 mg/kg, i.p.) were injected intraperitoneally whereas methyl-scopolamine bromide (a muscarinic receptor antagonist; RBI, USA, 1 mg/kg, s.c.) was given subcutaneously and used to prevent the peripheral cholinomimetic effects elicited by pilocarpine. All drugs were freshly dissolved in saline solution (NaCl 0.9%), which was used as control solution as well, in a volume injection of 1 ml/kg. All doses used here were taken from previous studies [5–7, 33].

### Behavioral test

#### Elevated plus-maze (EPM)

The EPM apparatus (EP151, Insight Ltda., Ribeirão Preto, Brazil) was made of wood and consisted of two opposing open arms (50 cm × 10 cm) and two opposing enclosed arms (50 cm × 10 cm × 30 cm) mounted at an angle of 90°, all facing a central platform (10 cm × 10 cm), elevated 40 cm from the floor. To prevent falls, the open arms were surrounded by a 1-cm high acrylic rim. The apparatus was placed in a small closed room lit by a 15-W red light that provided 3 lux in both the open and closed arms. Each rat was used only once and was placed individually on the central platform facing an enclosed arm. The frequency of entries into either open or enclosed arms as well as the time spent in each arm type were recorded (in seconds) for 5 min [39]. Ethological parameters such as protected stretch-attend postures, unprotected head-dipping, open-arms end activity and rearing were also recorded to increase the sensitivity of the test [40, 41]. The apparatus was cleaned with 10% ethanol solution between sessions.

### Experimental procedures

Experiments were performed in two main designs: In experiment 1, aiming to better understand hormonal properties of rats treated with pilocarpine, *ex vivo* biochemical assays were performed on blood samples (n = 3–6 per group) for CORT and adrenocorticotropic hormone (ACTH) quantification and samples from hippocampus (n = 6 per group) were extracted to analyze GR expression short (24 h) and long after treatment (1 month). In experiment 2, taking into account the pivotal role of NMDARs in the modulation of anxiety [26] and neural plasticity [42] following mAChR activation [43], samples from hippocampus were also extracted to analyze NMDARs expression in rats injected with saline or pilocarpine (n = 5–6 per group). Further, additional groups were previously treated with saline or memantine following 30 min later by a single systemic injection of saline or pilocarpine. Behavioral evaluation of these groups (n = 12 per group) was carried out in the EPM test 24 h or 1 month after treatments and the brain randomically extracted (n = 3–6 per group) immediately after the tests. Rats were euthanized and the brain was removed and washed with saline solution (NaCl 0.9%) at 4°C, dissected over a cooled Petri dish. The samples were stored in Eppendorfs and kept in a freezer at -80°C.

### Blood sampling and hormone measures

Twenty four hours or 1 month after the injection of pilocarpine, animals were housed in a sound-proof room for up to 48 h, prior to be euthanized with isoflurane and decapitated using a guillotine. Trunk blood was collected on ice into tubes containing 50 μl of EDTA (0.5 m; pH 7.4) and 50 μl of aprotinin (5000 KIU/ml, Trasylol; Bayer, EDTA, Newbury, UK). Plasma was separated by centrifugation and then stored at −80°C until processed for CORT and ACTH measurement. CORT levels were determined in triplicate by a double antibody radioimmunoassay method as previously described [44]. Antisera was kindly supplied by Prof. G. Makara (Institute of Experimental Medicine, Budapest, Hungary), and [125I] CORT was purchased from Izotop (Budapest, Hungary). The intra- and interassay coefficients of variation of the CORT assay were 16.7% and 13.3%, respectively. ACTH in plasma was measured using an immunoradiometric (IRMA) assay (DiaSorin, Stillwater, MN, USA) in accordance with the manufacturer’s instructions. The intra- and inter-assay coefficients of variation of the ACTH assay were 2.8% and 6.4%, respectively.

### Western Blotting

Rats were killed by decapitation 24 h or 1 month after treatment (immediately after the behavioral tests) and the hippocampus were removed and homogenized 1:10 (w/v) in HEPES 20 mM, pH 7.4 buffer, as previously described by Dutra *et al*., [45]. For Western blotting assays, the hippocampal tissue was homogenized in complete radioimmunoprecipitation lysis buffer (RIPA) containing 50 mM Tris-HCl (pH 7.2), 150 mM NaCl, 2 mM EDTA, 1% Igepal CA-630, 1 mM Na 3 VO 4, 50 mM NaF, 1 mM PMSF, 20 μg/mL pepstatin A, 20 μg/mL leupeptin and 20 μg/mL aprotinin. The lysate was centrifuged twice at 13,000 g for 10 min at 4°C, and the supernatant was collected for protein concentration determination and preparation for electrophoresis. Equal amounts of protein extract (20 μg) were loaded per lane and electrophoretically separated using 10% denaturing polyacrylamide gel electrophoresis (SDS-PAGE). Afterward, the proteins were transferred to nitrocellulose membranes using a Mini Trans-Blot Cell System (Bio-Rad Laboratories Inc., Hercules, CA, USA) following the manufacturer’s protocol. The membrane was blocked with 5% BSA in 0.05% TBST for 1 h at room temperature and then immunoblotted with the following antibodies: anti-β-actin (#3700, 1:1000, Cell Signaling Technology, Danvers, USA), anti-NMDAR1 (#4204, 1:1000, Cell Signaling Technology, Danvers, USA), anti-NMDAR2B (#MAB5220, 1:1000, EMD Millipore Corporation, Billerica, USA) and anti-GR (#SAB4501310, 1:500, Sigma-Aldrich) in blocking buffer at 4°C overnight. Following washing, the membranes were incubated with secondary antibodies conjugated to horseradish peroxidase (1:25,000, Cell Signaling Technology, Danvers, MA, USA). The immunocomplexes were visualized using SuperSignal West Femto Chemiluminescent Substrate Detection System (Thermo Fischer Scientific, Rockford, IL, USA) and densitometric values were normalized using β-actin densitometric values. Protein levels were quantified by optical density using Image-J Software® and expressed as the ratio to β-actin represented by arbitrary units.

### Statistical analysis

All values are expressed as means ± S.E.M. Data of experiments were analyzed by unpaired two-tailed Student´s *t* test when only the treatment factor was the grouping variable or ANOVA when two grouping variables–pretreatment and treatment–were used as factors, followed by the Student Newman–Keuls’ *post hoc* test for multiple comparisons when appropriate. Differences were considered significant at p ≤ 0.05. All tests were performed using the software Statistica® (StaSoft Inc.,Tulsa, USA), version 8.0 and graphs were drawn with the software GraphPad Prism®, version 5.0.

## Results

### Experiment 1: Distinct hormonal responses following mAChR activation

**Fig 1:**
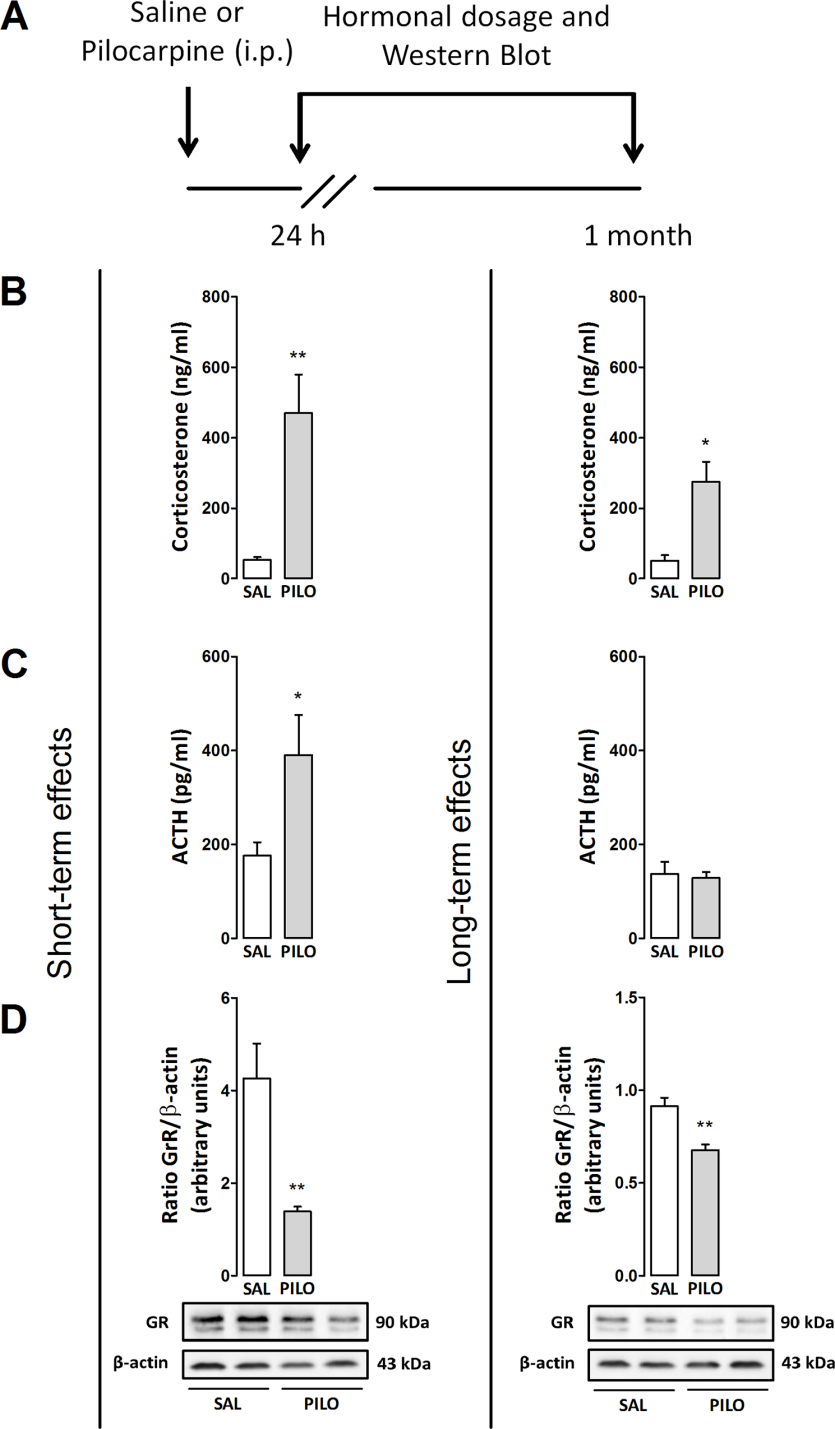
(A) Outline of the treatments and experimental schedule: saline (Sal, 0.9%) or pilocarpine (Pilo, 150 mg/kg). (B and C) Effects of pilocarpine on plasma levels of CORT (ng/ml) and ACTH (pg/ml) and (D) expression of GRs (85 and 117 kDa) in the hippocampus of rats injected with pilocarpine. Animals were euthanized 24 h or 1 month after treatments. Protein expression levels of GRs were determined by Western blotting analysis, quantified by densitometric scanning and expressed as a ratio of signal intensity for the target proteins relative to that for β-actin (43 kDa). Data are presented as mean±S.E.M. of 3–6 animals per group. Comparisons were made by Student´s t test. *p≤0.05 or **p<0.001 as compared with saline (control) group.

To better investigate the long-term effects induced by pilocarpine CORT and ACTH plasma levels were determined 24 h or 1 month after treatment (see experimental design in Fig 1A). As shown in Fig 1B–1D, the injection of pilocarpine significantly increased plasma CORT levels (unpaired t-test, t(9) = 3.43; p<0.001) and ACTH levels (unpaired t-test, t(9) = 2.58; p = 0.05) whereas the hippocampal expression of GR was significantly decreased (unpaired t-test, t(10) = 4.06; p<0.001) 24 h after the treatment when compared with control rats. Similarly, pilocarpine also increased plasma CORT levels 1 month after treatment (unpaired t-test, t(6) = 2.91; p<0.05) but not ACTH levels (p>0.05) besides decreased the hippocampal expression of GR (unpaired t-test, t(10) = 6.29; p<0.001) when compared with the control group, as observed in Fig 1B–1D.

### Experiment 2: The role of NMDARs on modulation of hippocampal plasticity and behavioral anxiety following mAChR activation

**Fig 2:**
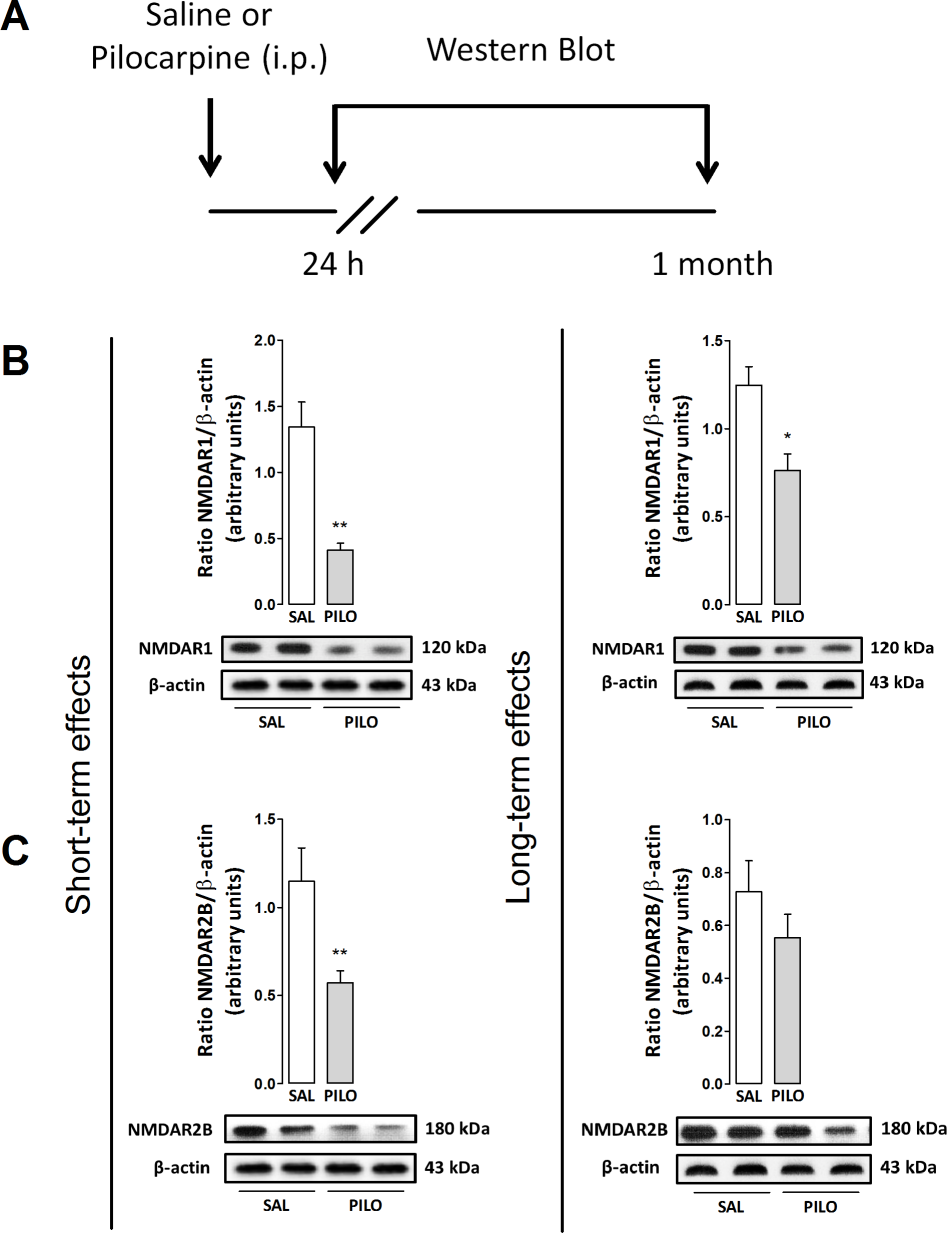
(A) Outline of the treatments and experimental schedule: saline (Sal, 0.9%) or pilocarpine (Pilo, 150 mg/kg). (B and C) Effects of pilocarpine on the expression of NMDAR1 (120 kDa) and R2B (180 kDa) in the hippocampus of rats. Animals were euthanized 24 h or 1 month after treatments. The protein expression levels of NMDARs were determined by Western blotting analysis, quantified by densitometric scanning and expressed as a ratio of signal intensity for the target proteins relative to that for β-actin (43 kDa). Data are presented as mean±S.E.M. of 5–6 animals per group. Comparisons were made by Student´s t test. *p≤0.05 or **p≤0.001 as compared with saline (control) group.

To investigate the role of NMDAR on long-term responses elicited by pilocarpine, we examined the expression of NMDAR excitatory subunits (NR1 and NR2B) in the hippocampus following treatment (Fig 2A). As shown in Fig 2B and 2C, pilocarpine significantly decreased the expression of NMDAR1 (unpaired t-test, t(9) = 5.15; p<0.001) and NMDAR2B (unpaired t-test, t(9) = 2.69; p<0.001) in rats measured 24 h after injection when compared with control groups. Additionally, a similar response was observed when the expression of NMDAR1 was quantified 1 month after pilocarpine (unpaired t-test, t(8) = 4.23; p<0.05) whereas no significant effect was observed when the expression of R2B was quantified (p>0.05), as observed in Fig 2.

**Fig 3:**
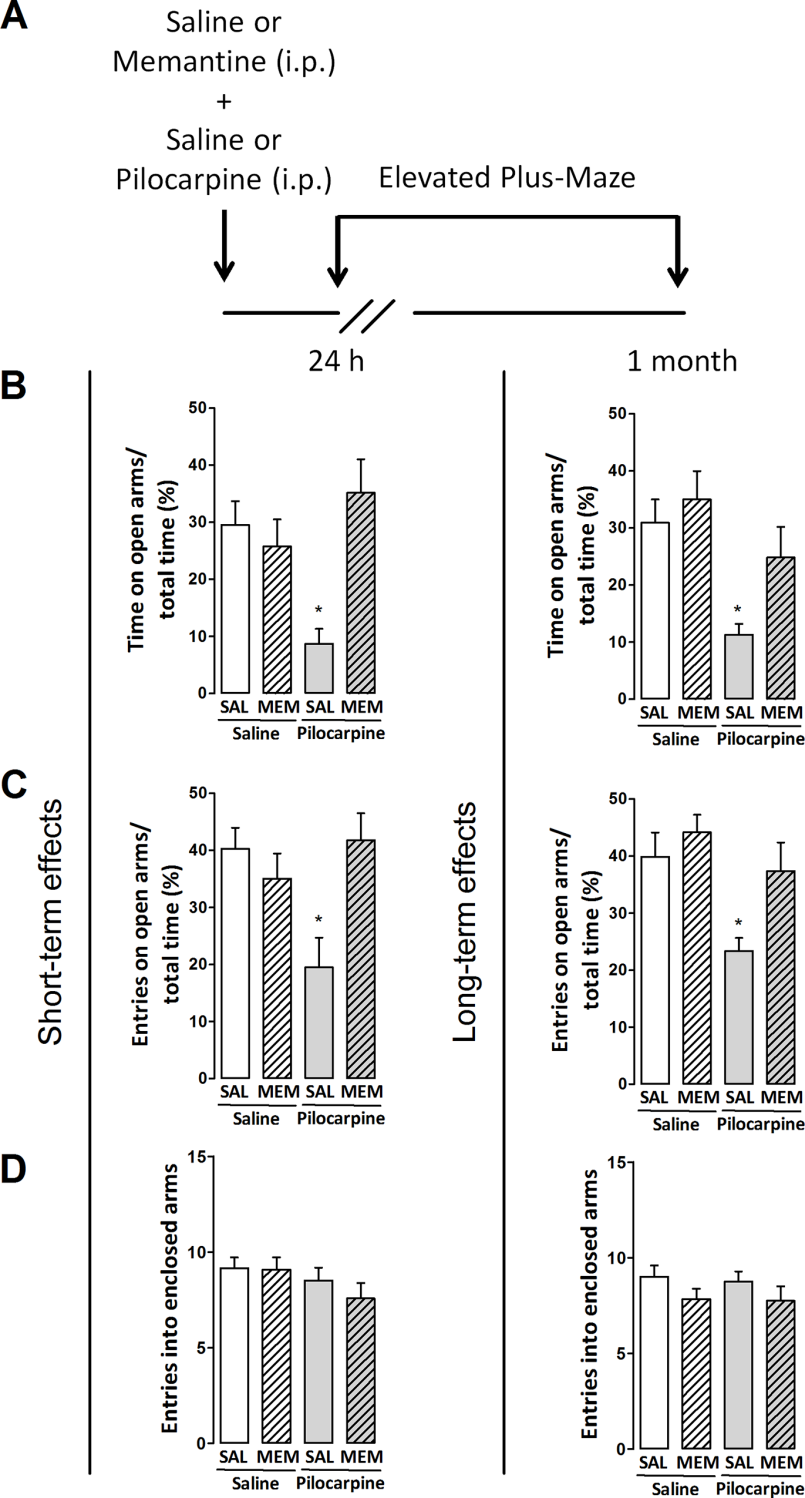
(A) Outline of the treatments and experimental schedule: memantine (Mem, 4 mg/kg), saline (Sal, 0.9%) or pilocarpine (150 mg/kg). (B–D) Effects of memantine on classic behavioral parameters of rats evaluated in the elevated plus-maze test 24 h or 1 month after treatments. Values are represented by the mean±S.E.M. of 12 animals per group. Comparisons were made by two-way ANOVA followed by Student–Newman–Keuls's test. *p≤0.05 as compared with Sal+Sal group.

To determine whether the effects of pilocarpine on altered hippocampal NMDAR expression were directly related to its long-term anxiogenic profile [5–7] we performed behavioral experiments in rats treated with the NMDAR antagonist memantine 30 min prior to pilocarpine, and evaluated 24 h or 1 month later in the EPM test (see treatment protocol in Fig 3A). The two-way ANOVA showed that pretreatment (F(1,44) = 6.3, p<0.05), treatment (F(1,44) = 1.6, p>0.05) and the interaction effect between these factors (F(1,44) = 11.3, p<0.05) can alter the performance of rats tested 24 h after injections, as observed in time spent in the open arms of the maze. Similarly, pretreatment (F(1,44) = 2.3, p>0.05) and treatment (F(1,44) = 3.4, p>0.05) show no effects, whereas the interaction between these factors demonstrate significant differences (F(1,44) = 9.3, p<0.05) when the number of open arms entries is evaluated in the maze. Furthermore, the number of entries into enclosed arms of the maze were not changed following two-way ANOVA (p>0.05). The *post hoc* analysis revealed that rats treated with saline and pilocarpine display an anxiogenic-like profile 24 h following treatment, as denoted by a decrease in the time spent in the open arms of the maze (p<0.05) and the number of open arms entries (p<0.05) whereas the number of entries into enclosed arms was unaffected (p>0.05). In addition, memantine effectively blocked behavioral pilocarpine-induced effects when compared with control groups (p>0.05, Fig 3).

Similar effects were observed in rats evaluated 1 month after injections as observed by two-way ANOVA showing that pretreatment (F(1,44) = 4.2, p<0.05) and treatment (F(1,44) = 11.9, p<0.05) but not the interaction effect between these factors (F(1,44) = 1.2, p>0.05) can alter the time spent in the open arms of the maze. In the same way, pretreatment (F(1,44) = 5.7, p<0.05) and treatment (F(1,44) = 9.3, p<0.05) but not the interaction effect between these factors (F(1,44) = 1.7, p>0.05) change the number of open arms entries of rats evaluated in the maze. Again, no differences between groups were observed when the number of entries into enclosed arms were quantified (p>0.05, Fig 3). The *post hoc* analysis revealed that rats treated with saline and pilocarpine also decreased the time spent in the open arms of the maze (p<0.05) and the number of open arms entries (p<0.05) whereas the number of entries into enclosed arms was unaffected (p>0.05) 1 month after treatment. Similarly, memantine pretreatment blocked the anxiogenic effects elicited by pilocarpine (p>0.05, Fig 3).

Memantine pretreatment was also able to prevent the anxiogenic-like ethologic behaviors observed after the treatment with pilocarpine. According with two-way ANOVA, the number of protected stretch-attend postures in the EPM test was not modified 24 h after pretreatment (F(1,44) = 0.03, p>0.05) and treatment (F(1,44) = 0.03, p>0.05) despite a significant effect on the interaction between these factors (F(1,44) = 4.4, p<0.05). The number of head dipping behavior events presented significant effects in pretreatment (F (1,44) = 8.6, p<0.05) but not in the treatment (F(1,44) = 0.01, p>0.05) whereas interaction between these factors was significantly altered (F(1,44) = 9.3, p<0.05). Further, non significances were observed in open-arms end activity following pretreatment (F(1,44) = 1.2, p>0.05), treatment (F(1,44) = 1.7, p>0.05) or interaction between factors (F(1,44) = 3.5, p>0.05) or rearing behaviors following pretreatment (F(1,44) = 0.03, p>0.05) and treatment (F(1,44) = 1.5, p>0.05), excepting the interaction between factors (F(1,44) = 5.1, p<0.05). *Post hoc* test did not reveal differences between groups when applied, apart from an increased number of head dipping behaviors in memantine-treated rats injected with pilocarpine (S1 Fig).

Moreover, two-way ANOVA revealed that in rats evaluated 1 month after the treatments the number of protected stretch-attend postures is affected by treatment (F(1,44) = 6.3, p<0.05) but not pretreatment (F(1,44) = 2, p>0.05) nor the interaction between these factors (F(1,44) = 2, p>0.05). Head dipping behavior was similarly affected by treatment (F(1,44) = 7.2, p<0.05) and not by pretreatment (F(1,44) = 1.3, p>0.05) or interaction between factors (F(1,44) = 0.6, p>0.05) as well as the number of open-arms end activity which was affected by treatment (F(1,44) = 9.1, p<0.05) but not pretreatment (F(1,44) = 2, p>0.05) or interaction between factors (F(1,44) = 0.6, p>0.05). No differences were observed when the number of rearings was analyzed (p>0.05, S1 Fig). The *post hoc* test revealed that rats treated with saline or memantine and pilocarpine showed an ethologic anxiogenic-like profile, as shown by an increase in the number of protected stretch-attend postures (p<0.05), decrease in the number of head dipping behavior events (p<0.05) and in open-arms end activity (p<0.05) whereas the number of rearing events was unaffected by treatments (p>0.05, S1 Fig). Important to note, memantine effectively blocked behavioral pilocarpine-induced effects when compared to control groups as no effective changes in ethologic behaviors were observed following treatments (p>0.05) excepting an increased number of head dipping behavior 24 h after treatment (S1 Fig).

**Fig 4:**
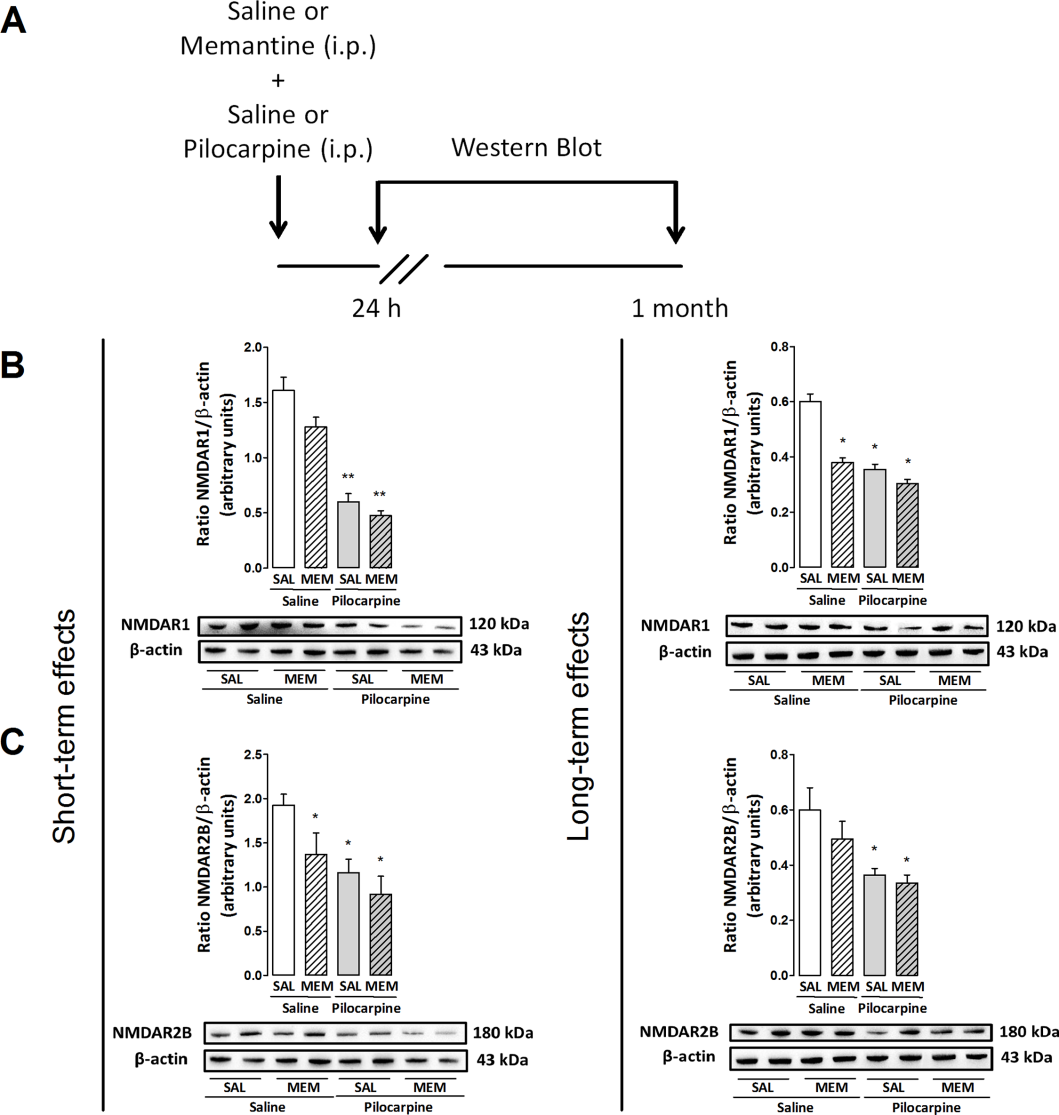
(A) Outline of the treatments and experimental schedule: memantine (Men, 4 mg/kg), saline (Sal, 0.9%) or pilocarpine (150 mg/kg). (B–C) Effects of memantine on the expression of NMDAR1 (120 kDa) and R2B (180 kDa) in the hippocampus of rats injected with pilocarpine. Animals were euthanized 24 h or 1 month after treatments. Protein expression levels of NMDARs were determined by Western blot analysis, quantified by densitometric scanning and expressed as a ratio of signal intensity for the target proteins relative to that for β-actin (43 kDa). Each group is represented by illustration of two lanes in order to denote intra-group variations. Data are presented as mean±S.E.M. of 3–6 animals per group. Comparisons were made by two-way ANOVA followed by Student–Newman–Keuls's test. *p≤0.05 or **p≤0.001 as compared with sal+sal group.

As the pretreatment with the NMDAR antagonist memantine was found to block pilocarpine-induced long term anxiogenesis in rats, we also determined whether these behavioral responses are related with the modulation of hippocampal NMDAR expression. In Fig 4, the expression of NMDAR1 were observed 24 h following ANOVA, with significant effects in pretreatment (F(1,8) = 6.9, p<0.05) and treatment (F(1,8) = 108.2, p<0.05) but not in the interaction between these factors (F(1,8) = 1.4, p>0.05). Similar responses were observed when the expression of NMDAR2B was quantified, with effects on pretreatment (F(1,20) = 4.6, p<0.05) and treatment (F(1,20) = 10.6, p<0.05) but not in the interaction of the factors (F(1,20) = 0.7, p>0.05). The *post hoc* test reveals that pilocarpine decreased the expression of NMDAR1 (p<0.001) and R2B (p<0.05) in rats pretreated with saline or memantine (Fig 4B and 4C). Likewise, NMDAR2B expression was reduced following 24 h after treatment with memantine and saline (p<0.05, Fig 4C).

In addition, Fig 4 reveals the expression of NMDAR1 1 month after injections with significant changes following pretreatment (F(1,8) = 47.3, p<0.05), treatment (F(1,8) = 66.8, p<0.05) and interaction between factors (F(1,8) = 18.4, p<0.05). Differently, the expression of R2B was affected by treatment (F(1,18) = 13.2, p<0.05) but not by pretreatment (F(1,18) = 1.6, p>0.05) or interaction between factors (F(1,18) = 0.5, p>0.05). The *post hoc* test shows that pilocarpine decreased NMDAR1 expression in rats pretreated with memantine or saline (p<0.05, Fig 4B). Similarly, memantine plus saline reduced hippocampal NMDAR1 expression in rats evaluated 1 month later (p<0.05, Fig 4B). Pilocarpine also induced a decrease in the expression of NMDAR2B of rats pretreated with either saline or memantine (p<0.05, Fig 4C).

## Discussion

In the present study, we used the non-selective cholinergic receptor agonist pilocarpine—previously known to experimentally induce long-term anxiety-like responses in rats [5–7]—to determine whether and how these effects are related to HPA-axis function and plastic changes of hippocampal NMDARs. We demonstrated that mAChR activation via pilocarpine disrupts HPA-axis activity, by changing not only the release of ACTH and CORT but also the expression of hippocampal GRs and NMDAR subunits. In addition, the pharmacological blockade of NMDARs with memantine inhibited anxiogenic-like behaviors induced by pilocarpine in rats, but did not prevent hippocampal NMDAR downregulation. Therefore, we suggest that a better understanding regarding the alterations of NMDAR subunits plasticity following mAChRs stimulation, and its role in anxiogenesis maintenance, may reveal a putative target to better comprehend the pathophysiology of emotional disorders.

It was demonstrated over the last years that mAChRs activated by a single non-convulsant injection of pilocarpine induces long-term anxiogenic-like behaviors in rats [5–7]. Here, we provide direct evidence that long-term behavioral anxiogenesis is also associated with lasting changes in HPA-axis function. In normal basal condition, CORT secretion is tightly regulated by the release of ACTH from the anterior pituitary. However, a dissociation between CORT and ACTH secretion has been observed in a number of conditions, including chronic stress and anxiety states [46]. In our experiments, we found both plasma CORT and ACTH levels markedly increased 24 h following pilocarpine injection, whereas only CORT levels remains elevated 1 month later. It is feasible that the occurrence of epigenetic factors regulating long-term CORT release occurs after mAChR activation, despite the return of ACTH levels to a normal status following one month. Similar alterations of HPA-axis function following a single exposure to stress in rodents (e.g. footshock, restraint in tubes) seems to occur predominantly by modifications of CORT rather than ACTH [47, 48], indicating a similar long-term ACTH-independent regulation of CORT release [49]. In addition, we hypothesize that this effect may also happen with an initial ACTH-mediated mechanism, resulting in increased sensitivity of the adrenal gland to ACTH, or via non-ACTH-mediated splanchnic neural activation of the adrenal [50, 51].

Hippocampal GRs were significantly decreased both early (24 h) and long after (1 month) pilocarpine treatment. These effects have parallels to studies in which rats exposed to a single prolonged stress also show decreased mineralocorticoid (MR)- and GR-immunoreactivity, and decreased expression of these receptors in the CA1 area of the hippocampus [52]. The increase of glucocorticoid levels during the acute phase following a stress stimulus result in adaptive responses which may cause negative effects on neural processing if hormonal levels remains high for a longer time, and may trigger cognitive impairments [53], neurotoxicity [54] as well as disrupting hippocampal negative feedback function over the HPA-axis [55]. Glucocorticoids may also alter hippocampal plasticity in association with NMDARs by modulating stress and atrophy of apical dendrites from hippocampal CA3 pyramidal neurons. In the same way, NMDAR-dependent long-term potentiation (LTP) is impaired by long-term stress, but blocked by RU486, a GR antagonist, suggesting a pivotal role of GRs following chronic stress [56].

As previously reported, mAChRs activation–specifically the M1 subtype–may potentiate excitatory transmission in the CNS through regulation of NMDARs [9] but the mechanisms involved in gain or loss of the NMDAR function are not yet clear. Several pathways converge toward the NMDARs modulating their gain, and thus the efficacy of synaptic transmission [43, 57]. In the present study, we showed that an anxiogenic dose of pilocarpine significantly downregulate NMDARs after treatment, suggesting a putative pathway which may modulate anxiety-like states in rats through plastic changes of the excitatory subunits R1 and R2B in the hippocampus. Unlikely, Di Maio and colleagues [58] reported that pilocarpine treatment induces early overexpression of hippocampal NMDARs in both *in vivo* and *in vitro* assays when measured 24 h after treatment, showing a critical involvement of inositol 1,4,5-trisphosphate (IP3) synthesis on NMDAR hyperactivation, and subsequent NADPH oxidase activation and NMDAR-independent ERK1/2 phosphorylation.

Important to note, the authors focused their work on better understanding early cellular responses during epileptogenesis, thus using higher doses of pilocarpine which are well known to experimentally induce seizures (for review see [59]). Similarly, NMDAR1s mRNA are increased in the hippocampus 50 days after pilocarpine-induced status epilepticus [60]. Aiming to avoid this bias regarding the occurrence of seizures triggered by pilocarpine, we previously showed that the effects of low doses of pilocarpine associated with the anxiogenic-like responses are not related with any electrographic or behavioral epileptiform activity aside from an increased hippocampal theta rhythm up to 1 month after treatment [6, 7]. Accordingly, it seems that seizure induction may trigger complex and divergent plastic alterations of NMDARs in hippocampal synapses.

The muscarinic system may itself play an indirect dual role controlling NMDARs plasticity in the hippocampus by opposing action of protein tyrosine kinases and phosphotyrosine phosphatases [61]. Activation of the M1 subunit of muscarinic receptors leads to phosphorylation and overexpression of NMDARs function in the CA3 area by activation of Src family of protein tyrosine kinases whereas the activation of M1 subunit may also activate a protein tyrosine phosphatase that dephosphorylate NMDARs, enabling the dual control of NMDAR expression [61]. According to Grishin and colleagues [57], mAChRs and metabotropic glutamatergic receptors can couple with two distinct pathways that stimulate or inhibit NMDAR function, depending on calcium concentration and cell-specific states. Therefore, our findings point to an important role of mAChRs activation on desensitization of NMDARs, thus possibly modulating HPA-axis regulation and eliciting anxiogenic-like effects after pilocarpine treatment, although a more detailed investigation regarding the downstream effects induced by mAChRs and NMDARs modulation, as well as the putative involvement of intracellular Ca^2+^ on pilocarpine effects, must be carried out.

Likewise, not only may mAChRs regulate NMDARs but activation of NMDARs may itself cause phosphorylation and desensitization of mAChRs, a regulatory mechanism where glutamatergic and cholinergic receptors can feedback to each other altering the threshold value of the transmission networks involved [62]. In this sense, the anxiolytic effects induced by NMDAR blockers are believed to be critically mediated by hippocampal NMDARs [26]. Besides, the concomitant or single pre-training injection of subeffective doses of an NMDAR antagonist and an M1 muscarinic antagonist could impair freezing behavior time during contextual fear conditioning test (a hippocampal-dependent task) but not on tone fear conditioning test (a hippocampal-independent task), highlighting the involvement of M1 subtype receptors on fear-related hippocampal-dependent learning and memory processes [63].

In order to better understand the role of NMDARs activation on pilocarpine-induced anxiogenesis and also the possible NMDARs subunits downregulation as a consequence of it, we treated rats with memantine—an NMDAR noncompetitive antagonist [64]—prior to pilocarpine injection. We found that a subeffective dose of memantine could block pilocarpine effects according with behavioral parameters observed in the EPM test, with no occurrence of locomotor deficits [65, 66], evidencing a significant mediation of NMDARs in the behavioral pilocarpine effects. Corroborating, genetically modified mice with complete loss of the NMDAR NR1 subunit in the granule cells of the dentate gyrus exhibit a normal LTP in hippocampal CA1 region and an anxiolytic-like profile when behaviorally evaluated [67]. Additionally, genetically modified mice in which NR2B subunit was postnatally ablated from pyramidal and granular cells of hippocampus also presented anxiolytic responses and short-term spatial working memory deficits [68].

The involvement of NMDAR signaling on behavioral long-term anxiety induced by pilocarpine comprises a substantial progress regarding the mechanisms underlying these effects. Interestingly, our study shows that memantine was not able to alter pilocarpine-induced NMDARs downregulation 24 h or 1 month after treatment. A previous study showed that both MK-801 (a selective noncompetitive NMDAR antagonist) and BAPTA (a Ca^2+^ chelator) could prevent NMDAR2B/NMDAR1s overexpression in the hippocampus of epileptic rats 24 h later, suggesting the influence of NMDA-Ca^2+^ signaling pathway on early biochemical NMDARs plasticity [58]. On the other hand, investigations revealed that MK-801 blocks virtually all NMDAR-mediated synaptic current after stimulus, due its use-dependent and irreversible features [69, 70], besides presenting higher affinity and slow off-rate when compared to memantine [71, 72]. Several high-affinity NMDAR antagonists present efficacy on receptor blockade but also toxicity and side effects [73, 74]. This is one of the reasons why we have chosen memantine to perform our studies. Further, memantine is often used due to its safety clinical profile, avoiding unwanted adverse side effects but allowing NMDARs physiological function, blocking excessive extrasynaptic NMDAR-mediated currents whereas sparing normal synaptic activity of autaptic hippocampal neurons [75].

It is worth mentioning that we cannot exclude the involvement of other limbic pathways and neurotransmitter systems on memantine effects. Demontis and colleagues [76] recently showed the ability of memantine to prevent imipramine withdrawal-induced dopamine receptor desensitization and depressive-like behavior of rats. Further, memantine can increase GABA and dopamine levels in the nucleus accumbens of rats submitted to deep brain stimulation, denoting the modulatory action of NMDARs in the dopamine release [77]. Similar results also shown by other studies revealing this dual relation and ensuing behavioral effects [78–80]. In this sense, the broad range of action elicited by NMDARs modulation may explain why memantine does not effectively reverse hippocampal NMDARs plasticity but prevents anxiogenic-like behaviors induced by pilocarpine.

## Conclusion

We hypothesized that (i) anxiogenic-like pilocarpine effects seems to occur under non-toxic conditions since it does not induce electrographic seizures [6, 7], hippocampal glutamate uptake and cell viability alterations [6] nor hippocampal neurodegeneration (unpublished data), enabling a physiological status which may support GRs and NMDARs plasticity during anxiogenesis; (ii) memantine injection on pilocarpine-treated rats failed to change NMDAR-induced downregulation since is possibly allowing Ca^2+^ influx into hippocampal cells through NMDARs, a critical mediator on synaptic plasticity [57]; (iii) downregulation of NMDAR from pyramidal cells may occur through a Ca^2+^-dependent pathway activated after mAChR stimulation, which releases calcium ions from intracellular stores and possibly regulates NMDAR expression [43].

Here we have provided evidence that behavioral anxiogenesis induced by mAChR activation may trigger long-lasting effects on HPA stress-hormone release and on hippocampal GRs of rats. The activation of mAChRs effectively yields short- and long-term alterations in hippocampal NMDARs expression which possibly impair hippocampal inhibitory regulation over the HPA-axis. Although extrasynaptic NMDAR-mediated current blockade may prevent behavioral anxiogenesis, we have shown that these effects do not reflect a hippocampal NMDARs downregulation induced by pilocarpine. Altogether, our data provide new evidence for downstream biochemical phenomena that regulates anxiety-like responses in rats and highlights the muscarinic system as a target to future translational studies in this field.

## Supporting Information

S1 Fig (A) Outline of the treatments and experimental schedule: memantine (Mem, 4 mg/kg), saline (Sal, 0.9%) or pilocarpine (150 mg/kg). (B–E) Effects of memantine on ethologic behavioral parameters of rats evaluated in the elevated plus-maze test 24 h or 1 month after treatments. Values are represented by the mean±S.E.M. of 12 animals per group. Comparisons were made by two-way ANOVA followed by Student–Newman–Keuls's test. *p≤0.05 as compared with Sal+Sal group.
(TIF)

## Abbreviations

mAChR: muscarinic acetylcholine receptor
NMDAR: N-methyl-D-aspartic acid receptor
HPA axis: hypothalamic-pituitary-adrenal axis
CORT: corticosterone
ACTH: adrenocorticotropic hormone
MR: mineralocorticoid receptor
GR: glucocorticoid receptor
EPM: elevated plus-maze
