# Supplementary figures and images for: The Role of Hippocampal NMDA Receptors in Long-Term Emotional Responses following Muscarinic Receptor Activation
Source: PLoS One. 2016 Jan 21;11(1):e0147293. doi: 10.1371/journal.pone.0147293 (PMC4721870; doi:10.1371/journal.pone.0147293)

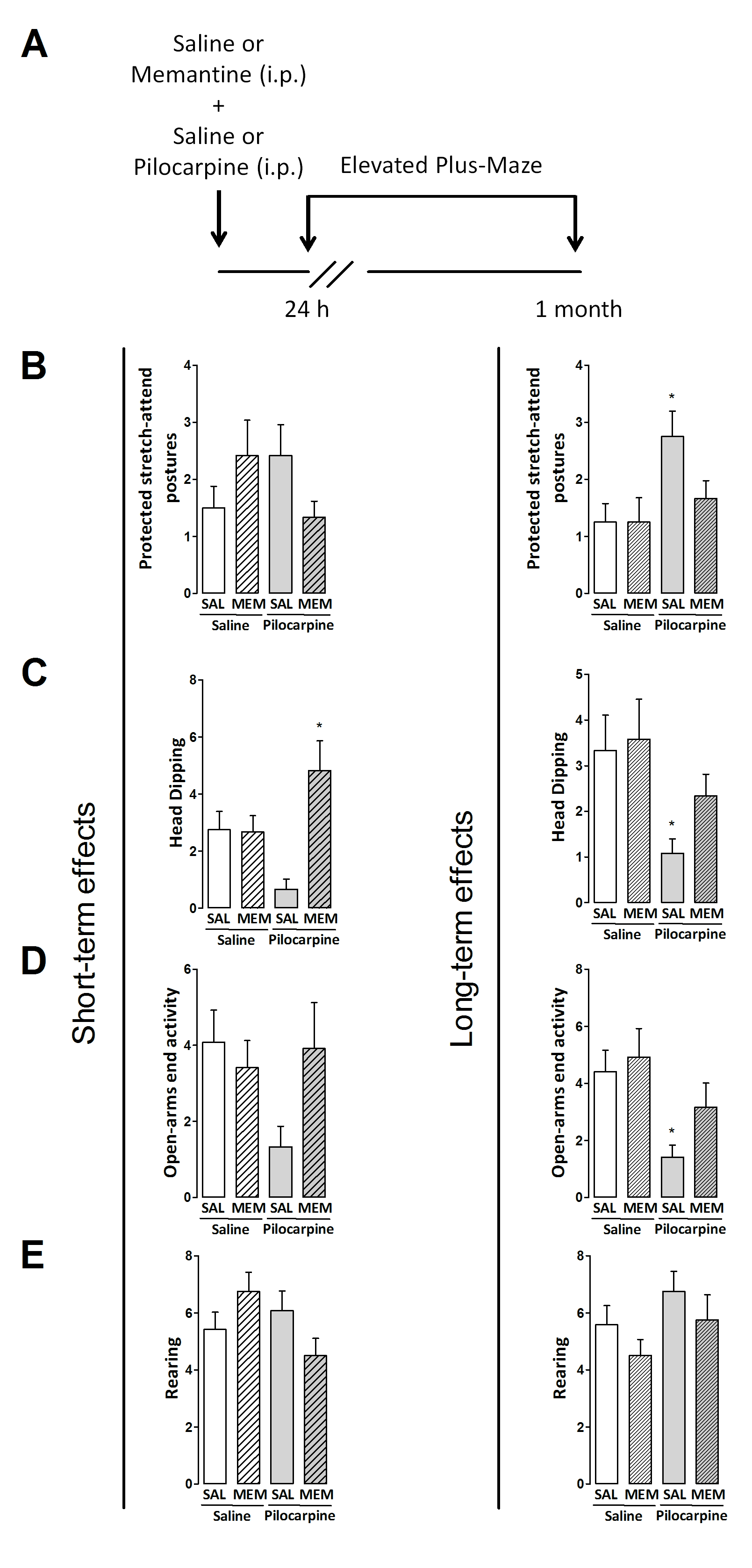

Supplement: S1 Fig — Values are represented by the mean±S.E.M. of 12 animals per group. Comparisons were made by two-way ANOVA followed by Student–Newman–Keuls's test. *p≤0.05 as compared with Sal+Sal group. (TIF) [file pone.0147293.s001.tif]
